# *Marsdenia tenacissima* extract induces apoptosis and suppresses autophagy through ERK activation in lung cancer cells

**DOI:** 10.1186/s12935-018-0646-4

**Published:** 2018-09-27

**Authors:** Yan-Na Jiao, Li-Na Wu, Dong Xue, Xi-Juan Liu, Zhi-Hua Tian, Shan-Tong Jiang, Shu-Yan Han, Ping-Ping Li

**Affiliations:** 1 Key Laboratory of Carcinogenesis and Translational Research (Ministry of Education/Beijing), Department of Integration of Chinese and Western Medicine, Peking University Cancer Hospital & Institute, No. 52 Fucheng Road, Haidian District, Beijing, 100142 People’s Republic of China; 2 Key Laboratory of Carcinogenesis and Translational Research (Ministry of Education/Beijing), Central Laboratory, Peking University Cancer Hospital and Institute, Beijing, 100142 People’s Republic of China

**Keywords:** *Marsdenia tenacissima* extract (MTE), Apoptosis, Autophagy, ERK activation, NSCLC

## Abstract

**Background:**

*Marsdenia tenacissima* is an herb medicine which has been utilized to treat malignant diseases for decades. The *M. tenacissima* extract (MTE) shows significant anti-proliferation activity against non-small cell lung cancer (NSCLC) cells, but the underlying mechanisms remain unclear. In this study, we explored the potential anti-proliferation mechanisms of MTE in NSCLC cells in relation to apoptosis as well as autophagy, which are two critical forms to control cancer cell survival and death.

**Methods:**

The proliferation of H1975 and A549 cells was evaluated by MTT assay. Cell apoptosis was assessed by Annexin V and PI staining, Caspase 3 expression and activity. Autophagy flux proteins were detected by Western blot with or without autophagy inducer and inhibitor. Endogenous LC3-II puncta and LysoTracker staining were monitored by confocal microscopy. The formation of autophagic vacuoles was measured by acridine orange staining. ERK is a crucial molecule to interplay with cell autophagy and apoptosis. The role of ERK on cell apoptosis and autophagy influenced by MTE was determined in the presence of MEK/ERK inhibitor U0126.

**Results:**

The significant growth inhibition and apoptosis induction were observed in MTE treated NSCLC cells. MTE induced cell apoptosis coexisted with elevated Caspase 3 activity. MTE also impaired autophagic flux by upregulated LC3-II and p62 expression. Autophagy inducer EBSS could not abolish the impaired autophagic flux by MTE, while it was augmented in the presence of autophagy inhibitor Baf A1. The autophagosome–lysosome fusion was blocked by MTE via affecting lysosome function as evidenced by decreased expression of LAMP1 and Cathepsin B. The molecule ERK became hyperactivated after MTE treatment, but the MEK/ERK inhibitor U0126 abrogated autophagy inhibition and apoptosis induction caused by MTE, suggested that ERK signaling pathways partially contributed to cell death caused by MTE.

**Conclusion:**

Our results demonstrate that MTE caused apoptosis induction as well as autophagy inhibition in NSCLC cells. The activated ERK is partially associated with NSCLC apoptotic and autophagic cell death in response to MTE treatment. The present findings reveal new mechanisms for the anti-tumor activity of MTE against NSCLC.

**Electronic supplementary material:**

The online version of this article (10.1186/s12935-018-0646-4) contains supplementary material, which is available to authorized users.

## Background

Lung cancer remains one of the leading causes of cancer-related deaths worldwide. It can be divided into small-cell lung cancer (SCLC, 15%) and non-small cell lung cancer (NSCLC, 85%) according to the histologic features. In patients with advanced NSCLC who generally have a poor prognosis [1], new strategies to improve survival are urgently required.

Aberrant signal transduction pathways often occur in tumorigenesis and progress. Studies demonstrated that autophagy and apoptosis play central roles during lung cancer initiation and progression [2]. Fundamental cellular physiological activities such as apoptosis and autophagy are critical to control cell survival and cell death [2]. Apoptosis is one form of programmed cell death with the function of removing damaged cells. Resistance to apoptosis is regarded as one of the hallmarks of cancer [3], thus targeting apoptosis in cancer is a practicable therapy with the suggest of many studies [4].

Autophagy is a self-degradation process to keep constant supply of cellular energy [5]. The relationship between autophagy and cell death is subtle and intricate, and it may promote or inhibit cell death in different contexts. The role of autophagy in tumor initiation and progression is multifaceted and complicated. It has been reported that autophagy inhibits tumorigenesis in some circumstances but promotes carcinogenesis under most conditions [6]. Through upregulating autophagy, cancer cells can survive, growth and become aggressive under pressured microenvironment [6]. Therefore, it makes autophagy as an attractive therapeutic target for effective treatment of tumors including lung cancer [7, 8].

Traditional Chinese Medicine has been used extensively to treat diseases from ancient time. The stem of *Marsdenia tenacissima* (Roxb.) Wight et Arn. is mainly produced in Yunnan (China), and its medical use was firstly recorded in “Dian Nan Ben Cao”, a medical literature written by Mao Lan in Ming Dynasty with the activity of expectorant, diuresis, eliminating heat and purging fire, lactating. *M. tenacissima* has long been used as a remedy to treat malignant diseases, tracheitis, and pneumonia in China [9, 10].

There is a great number of studies demonstrated that the water extract of *M. tenacissima* (MTE, trade name: Xiao-Ai-Ping injection) has anti-tumor effects in cell culture models, laboratory animal models and the clinics. (a) The cell culture models include gastric carcinoma cells (SGC-7901) [11], non-small cell lung cancer cells (H1975, H292, H460) [12], Burkitt lymphoma cells [13], human umbilical vein endothelial cells (HUVECs) [14, 15], hepatoma cells (HepG2) [14], esophageal cancer cells (KYSE150 and Eca-109) [16], etc. (b) Xenograft mouse models were generated from gastric cancer [11], hepatocellular carcinoma [17], lymphoma [13] and the chick embryo chorioallantoic membrane [14] etc. (c) The clinic trials were mainly conducted in advanced non-small cell lung cancer patients [18, 19]. Mechanisms accounting for the anti-tumor activities of MTE comprise of anti-angiogenesis [14], cell apoptosis induction [20] and cell cycle arrest [16]. However, the molecular mechanisms underlying the pharmacological action of MTE treatment resulting in cell death remains obscure and need further exploration.

Due to the vital role of apoptosis and autophagy in cell death, in the present study, we evaluated the influence of MTE on cell apoptosis and autophagy in NSCLC cell lines A549 and H1975. Meanwhile, the molecular mechanisms of MTE treatment shared by both apoptosis and autophagy were also explored and elucidated.

## Materials and methods

### Cell cultures and reagents

Human lung carcinoma cell lines A549 and H1975 (American Type Culture Collection, Manassas, VA, USA) were maintained in RPMI-1640 (GIBCO, Thermo Fisher, Hudson, NH, USA) supplemented with 10% fetal bovine serum, 100 U/ml penicillin and 100 μg/ml streptomycin in a humidified incubator at 37 °C under 5% CO_2_/95% air. The reagents used in this study were: Earle’s balanced salt solution (EBSS, Solarbio, H2020, Beijing, China), Bafilomycin A1 (Baf A1, B1793, Sigma-Aldrich, St. Louis, MO, USA), 3-(4,5-dimethyl-2-thiazolyl)-2,5-diphenyl-2-*H*-tetrazolium bromide (MTT, M2128, Sigma-Aldrich), 2-(4-amidinophenyl)-6-indolecarbamidine dihydrochloride (DAPI, D9542, Sigma-Aldrich), LysoTracker Red (L7528, Thermo Fisher), U0126 (S1102, Selleckchem, Houston, TX, USA). Antibodies of anti-LC3-II (L7543) was purchased from Sigma-Aldrich; anti-p62 (ab56416), anti-Cathepsin B (ab33538), and anti-Caspase 3 (ab32351) antibodies were purchased from Abcam (Cambridge, UK); anti-Poly (ADP-ribose) polymerase (PARP) (9542), anti-Lysosome-associated membrane protein 1 (LAMP1) (9091), anti-p-ERK1/2 (Thr202/Tyr204) (9101), anti- ERK1/2 (9102), anti-Bcl-2 alpha (2876) and anti-Bax (5023) were obtained from Cell Signal Technology (Beverly, MA, USA). Anti-β-actin (TDY041) was obtained from TDYbio (Beijing, China). Secondary antibodies including peroxidase-conjugated goat anti-mouse IgG (ZB2305), peroxidase-conjugated goat anti-rabbit IgG (ZB2301), and fluorescein-conjugated affiniPure goat anti-mouse IgG (ZF0312) were purchased from Beijing Zhongshan Golden Bridge Biotechnology Co. Ltd.

MTE (*M. tenacissima* extract, trade name: Xiao-Ai-Ping injection) (1 g crude/ml) was obtained from SanHome Pharmaceutical Co., Ltd (Nanjing, China). The stem of *M. tenacissima* was collected from Yunnan, China. A voucher specimen (200907-T009-05) was deposited in the herbarium of SanHome Pharmaceutical Co., Ltd (NanJing, China) and was identified by Professor De-Kang Wu (Nanjing University of Chinese Medicine). The preparation of MTE is described as previously [21]. 1 kg powder of the stem of *M. tenacissima* was extracted with water for three times which is 1.5 h, 1 h and 0.8 h, respectively. The combined extracts were filtered, concentrated, and then precipitated with 8 times 85% ethanol at 4 °C for 24 h. The ethanol was recovered and new 85% ethanol was added to cause further precipitation. The ethanol in extract was recovered thoroughly and the insoluble precipitate was removed by filtration. Finally, the extract was concentrating to 200 ml. This condensed extract was dilute with water for injection, added 0.3% polysorbate 80, and adjust pH to 5.5–6.0 to get Xiao-Ai-Ping injection following the standard of State Food and Drug Administration (SFDA) of China.

### Cell viability assays

A549 or H1975 cells were suspended in complete RPMI-1640 medium and plated at a density of 5 × 10^3^ cells/well in 96-well culture dishes (Costar, Cambridge, MA, USA). Following 24 h of culture, the medium was replaced with complete culture medium supplemented with various concentrations of drugs. On the collection time points, cells were incubated with MTT at 37 °C for 4 h, and the precipitate was dissolved in DMSO. Subsequently, the absorbance (optical density, OD) at 570 nm was measured using a microplate reader (Model 680; Bio-Rad Laboratories, Hercules, CA, USA) and cell viability was calculated according to the following formula: (OD_sample_ − OD_blank_)/(OD_control_ − OD_blank_) × 100%.

### Western blot analysis

For immunoblot analysis, cells were harvested and lysed in RIPA lysis buffer (WB0002, TDYBio). Protein concentrations were determined using the BCA protein assay kit (Thermo Fisher). Protein samples (20 μg per lane) were separated on the 8–15% SDS–polyacrylamide gel electrophoresis (PAGE) and blotted onto polyvinylidene difluoride membranes (Immobilon-P, Millipore, Bedford, MA, USA). Following transfer, the membranes were blocked in 5% nonfat milk or bovine serum albumin (BSA) (for phosphorylated proteins) in phosphate-buffered saline (PBS) with 0.1% Tween-20, probed with primary antibodies overnight at 4 °C. After washing, the membranes were incubated with appropriate horseradish peroxidase-conjugated secondary antibodies. Visualization of the protein bands was accomplished using an Immobilon Western Chemiluminescent HRP substrate (Millipore). Image J software was used to calculate the expression of each protein, which was normalized by β-actin.

### Apoptosis analysis

Cell apoptosis was assayed by Annexin V and PI staining (AD10, Dojindo, Kumamoto, Japan). Cells were treated with different concentrations of MTE for 24 h, without or with MEK/ERK inhibitor U0126 (50 µM for A549, 20 µM for H1975). Then cells were collected and incubated with the buffer containing FITC-conjugated Annexin V and PI for 15 min at room temperature, and then analyzed by FACScan flow cytometry (Bection Dikinson, USA). Quantification of early apoptotic cells (Annexin V^+^/PI^−^ cells) and late apoptotic cells (Annexin V^+^/PI^+^ cells) was calculated by CellQuest software.

### Caspase 3 activity assay

The activity of Caspase 3 was determined using a kit from Beyotime Institute of Biotechnology (C1116, Beijing, China). The activity of Caspase 3 was based on its ability to change acetyl-Asp-Glu-Val-Asp p-nitroanilide (Ac-DEVD-pNA) into a yellow formazan product (p-nitroaniline (pNA)). An increase in absorbance at 405 nm was used to quantify Caspase 3 activity. After 24 h exposure, cells with various designated treatments were collected and rinsed with cold PBS, and then lysed by lysis buffer (60 μL) for 15 min on ice, respectively. Cell lysates were centrifuged at 16,000×*g* for 15 min at 4 °C. The detail analysis procedure was described in the manufacturer’s protocol. The Caspase 3 activity was shown as fold change of enzyme activity compared to control. All the experiments were carried out in triplicates.

### Immunofluorescence, fluorescence, and confocal microscopy

Cells were seeded to cover glasses in 24-well plates and treated as indicated, fixed with 4% paraformaldehyde and permeabilized with 0.2% Triton X-100 (ST795, Beyotime). The cells were then blocked with 5% FBS for 1 h and exposed to anti-LC3-II (PM036, MBL, Nagoya, Japan) antibody overnight at 4 °C. After washing three times with PBS, cells were incubated with FITC-conjugated secondary antibody solution. After staining nuclei with DAPI, cells were observed under a confocal microscope (Leica, Welzler, Germany). For each group, the number of endogenous LC3-II puncta per cell was assessed in 100 cells, and statistical data were obtained from three independent experiments.

For LysoTracker staining, A549 or H1975 cells with stable expression of GFP-LC3 were cultured in confocal dishes and incubated for 90 min in complete RPMI-1640 medium supplemented with 500 nM LysoTracker Red. The colocalization of LC3 and LysoTracker was analyzed by the confocal microscopy.

### Acridine orange (AO) staining

Autophagy is a lysosomal degradation pathway for cytoplasmic material and organelles. The acidic intracellular compartments were visualized by supravital AO staining. After the treatment with MTE (0, 20, 40 mg/ml) for 6 h, cells were washed with PBS and stained with 1 μg/ml AO (318337, Sigma-Aldrich) for 20 min at 37 °C. Subsequently, cells were analyzed under the confocal microscopy.

### Statistical analysis

All experiments were repeated at least three times and values are expressed as the mean ± standard error of mean (SEM). Student’s t-test was used to determine the difference between two independent groups. All data were analyzed using SPSS statistical software 16.0 (SPSS Inc., Chicago, IL, USA). *P *< 0.05 was considered statistically significant difference between values.

## Results

### MTE suppressed lung cancer cell proliferation in vitro

**Fig. 1:**
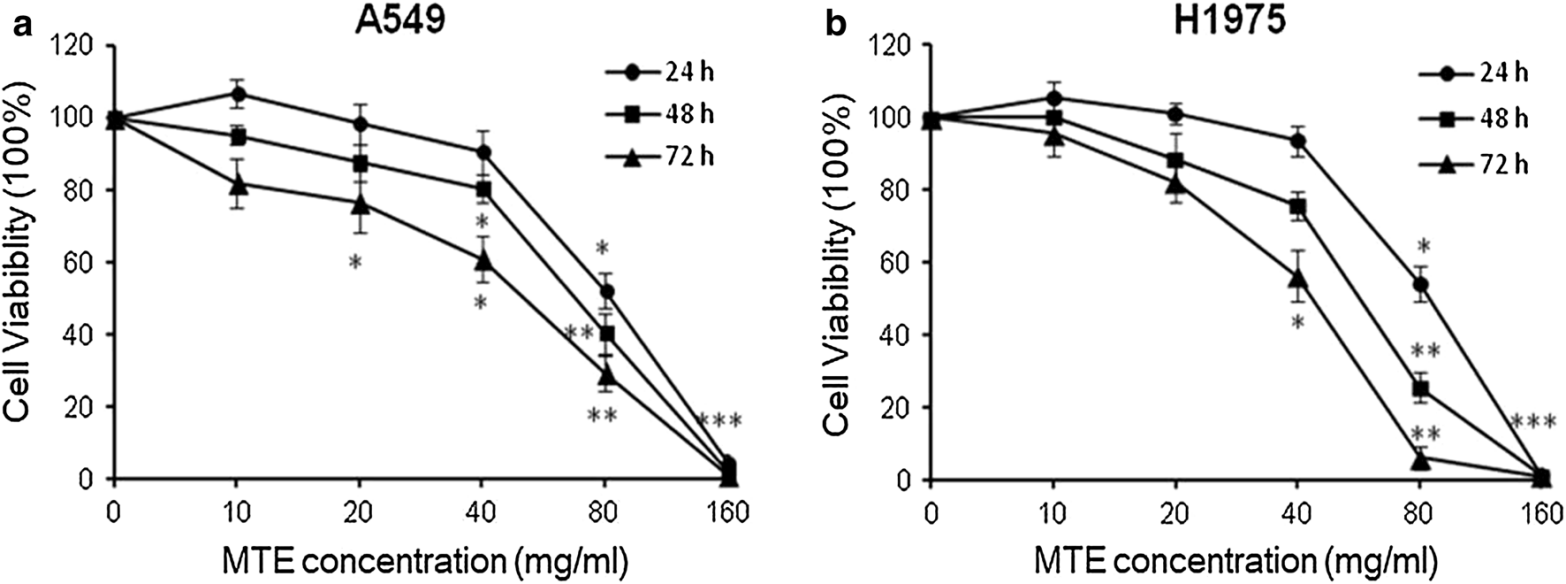
MTE suppressed lung cancer cell proliferation in vitro. MTT assay was performed to detect IC50 of MTE after 24, 48 and 72 h treatment in **a** A549 cells; **b** H1975 cells. Data were represented as mean ± SEM from three independent experiments. *P < 0.05, **P < 0.01, ***P < 0.005 vs control group

MTE is a Chinese medicine used to treat lung cancer, gastric cancer and other cancers with a good therapeutic efficacy. Firstly, we examined the IC50 (half maximal inhibitory concentration) in A549 and H1975 using MTT assay. The results demonstrated that MTE significantly inhibited NSCLC cells proliferation in a dose-dependent manner after 24, 48 and 72 h treatment. The IC50 values of A549 cells were 92.5 ± 4.3 mg/ml at 24 h, 69.0 ± 4.8 mg/ml at 48 h and 48.9 ± 5.1 mg/ml at 72 h, separately (Fig. 1a). The IC50 values of H1975 cells were 82.5 ± 4.9 mg/ml at 24 h, 56.3 ± 6.2 mg/ml at 48 h and 40.5 ± 3.0 mg/ml at 72 h, respectively (Fig. 1b). Such findings demonstrated that MTE can significantly suppress the growth of NSCLC cells.

### MTE treatment induced apoptosis in lung cancer cells

**Fig. 2:**
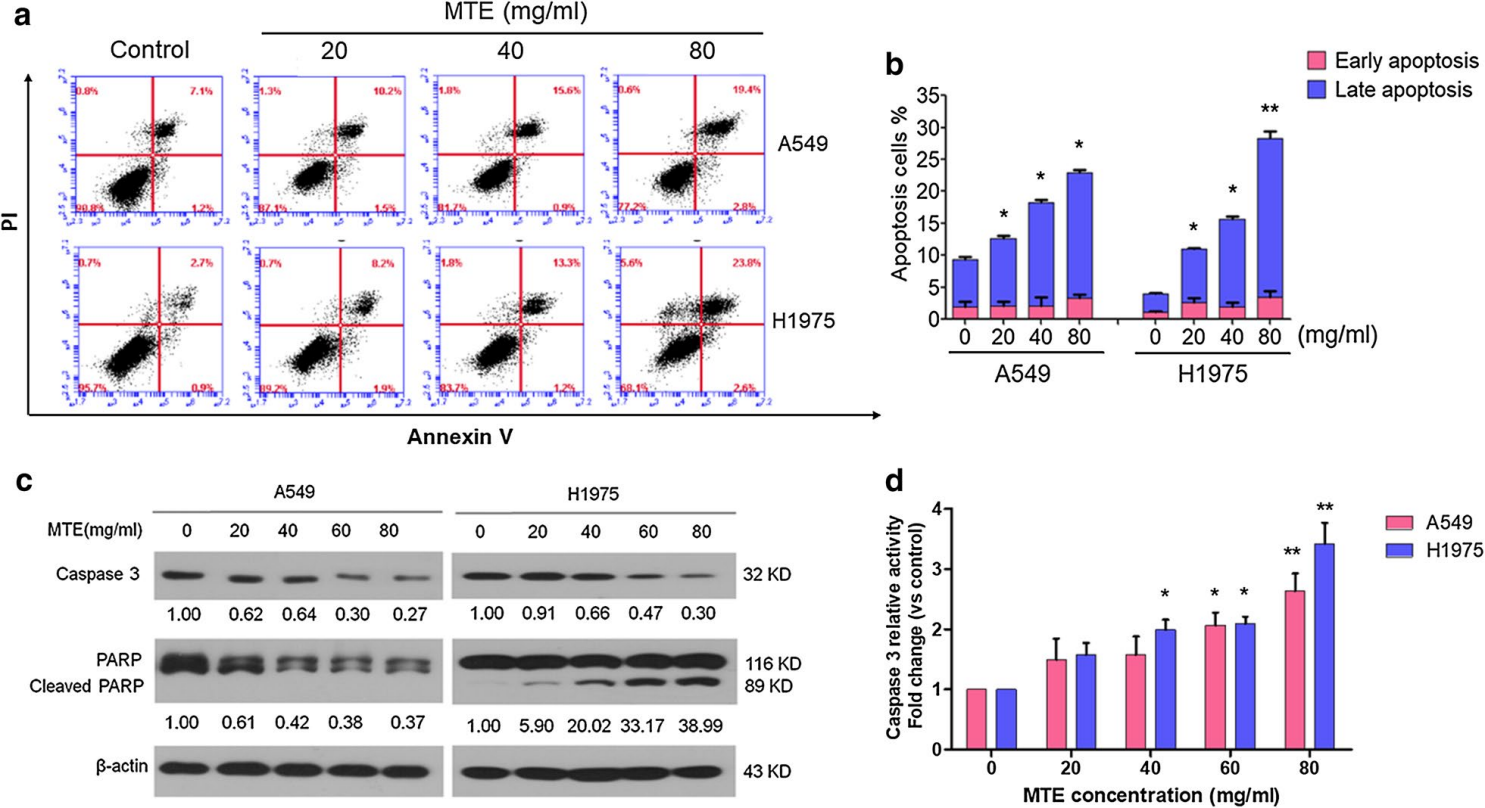
MTE treatment induced apoptosis in NSCLC cells. A549 and H1975 cells were treated with 0, 20, 40, 60 and 80 mg/ml MTE for 24 h. **a** Apoptotic cells were counted by Annexin V/PI assay. In the four fields of the original images, the dots indicated the number of Annexin V^−^/PI^−^ (bottom-left field indicates live cells), Annexin V^+^/PI^−^ (bottom-right field indicates early apoptotic cells), Annexin V^+^/PI^+^ (top-right field indicates late apoptotic cells), and Annexin V^−^/PI^+^ cells (top-left field indicates dead cells), respectively. **b** The percentage of early and late apoptotic cells were quantified, respectively. Early-stage apoptotic cells in the figures were shown in red, and late-stage apoptotic cells were shown in blue. **c** The protein levels of Caspase 3 and PARP were determined by Western blot, and the expression ratio was counted. β-Actin was used as loading control. Data are one representative experiment performed in triplicate. **d** The Caspase 3 activities in MTE treated cells were detected. A549 cells were shown in red, and H1975 cells were shown in blue. *P < 0.05, **P < 0.01 vs control group

To detect whether cell growth suppression after MTE treatment was through apoptosis, flow cytometry analysis was performed. As shown in Fig. 2a, b, NSCLC cells treated with various doses of MTE for 24 h caused cell apoptosis in a dose-dependent manner, especially in late-stage apoptosis. Briefly, MTE treatments led to cell late apoptotic rate from 7.5 ± 0.5% of control group to 10.6 ± 0.5% (20 mg/ml), 16.1 ± 0.7% (40 mg/ml) and 19.7 ± 0.4% (80 mg/ml) in A549 cells; from 2.9 ± 0.2% of control group to 8.4 ± 0.3% (20 mg/ml), 13.8 ± 0.6% (40 mg/ml) and 24.9 ± 1.5% (80 mg/ml) in H1975 cells. Meanwhile, the early apoptotic rate of cells in each group only ranged from 2.0 ± 1.1% to 3.3 ± 0.7% in A549, and from 1.1 ± 0.3% to 3.5 ± 1.3% in H1975.

Next, proteins associated with apoptosis were examined by Western blot. Treatment of MTE for 24 h decreased Caspase 3 zymogens expression (Fig. 2c) and increased Caspase 3 activities (Fig. 2d) in both cell lines. However, the cleaved PARP was raised in H1975 cells after MTE treatment, while only non-active PARP was reduced in A549 cells. In addition, mitochondrial associated apoptosis was involved in MTE-induced apoptosis, as evidenced by increased Bax and declined Bcl-2 protein expression (Additional file 1: Fig. S1A, B). These data indicated that cell apoptosis may contribute to cell growth suppression by MTE in NSCLC.

### MTE treatment disrupted autophagic flux in NSCLC cells

**Fig. 3:**
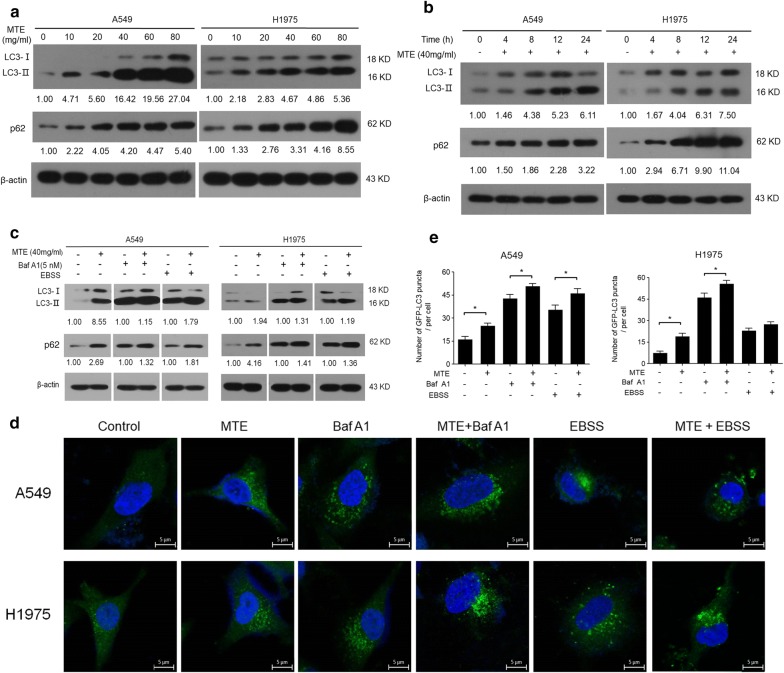
MTE treatment disrupted autophagic flux in NSCLC cells. **a**, **c** The protein levels of LC3-II and p62 in treated cells were determined by Western blot, and the ratio of protein levels was counted. **a** Cells were treated with MTE at the concentration of 0, 10, 20, 40, 60 and 80 mg/ml, respectively. **b** Cells were treated with MTE at 0, 4, 8, 12 and 24 h, respectively. **c** Cells were pretreated with 40 mg/ml MTE for 24 h following by Baf A1 (5 nM) or EBSS for another 4 h. β-Actin was used as loading control. **d** Endogenous LC3 levels (green fluorescence) were visualized on confocal microscope by immunofluorescence staining. **e** LC3 dots in A549 and H1975 cells were counted. Data represents mean ± SEM of at least 100 cells scored. *P < 0.05 for compared two groups

Recent reports showed that cell apoptosis and autophagy are often affected by anticancer agents [22]. In order to investigate the effect of MTE on autophagy, firstly we monitored the classic autophagic marker LC3-II by Western blot. As shown in Fig. 3a, b, LC3-II increased in a dose- and time-dependent manner after MTE treatment in both A549 and H1975 cells, indicating MTE influenced the process of cell autophagy. MTE treatment also raised p62 protein level in a dose- and time-dependent manner (Fig. 3a, b). p62 is an adaptor protein that serves as a link between LC3 and ubiquitinated substrates; its increase suggested the substrate degradation was blocked and the autophagic flux was impaired after MTE treatment.

The autophagy flux after MTE treatment was further determined through the addition of autophagy inducer EBSS or inhibitor Baf A1. As shown in Fig. 3c (lane 6 vs lane 5), compared with EBSS alone, the co-treatment of MTE exerted an enhanced increase of LC3-II and p62 expression in A549 and H1975 cells, indicating MTE still suppressed cell autophagy even concurrent with EBSS treatment. Moreover, a synergistically impaired autophagy flux was observed with the combined treatment of MTE and Baf A1, which is an inhibitor to block the fusion of autophagosome with lysosome (Fig. 3c, lane 4 vs lane 3). In addition, we monitored LC3-II puncta in cells by immunofluorescence. In Fig. 3d, e, compared with control group, MTE treatment augmented the LC3-II puncta distribution in the presence or absence of EBSS or Baf A1, which is similar to the results of Western blot. These data suggested that the increased LC3-II and p62 in MTE treated NSCLC cells were due to suppression of autophagic flux in the late stage of autophagy.

### MTE suppressed autophagy by affecting lysosomal function

**Fig. 4:**
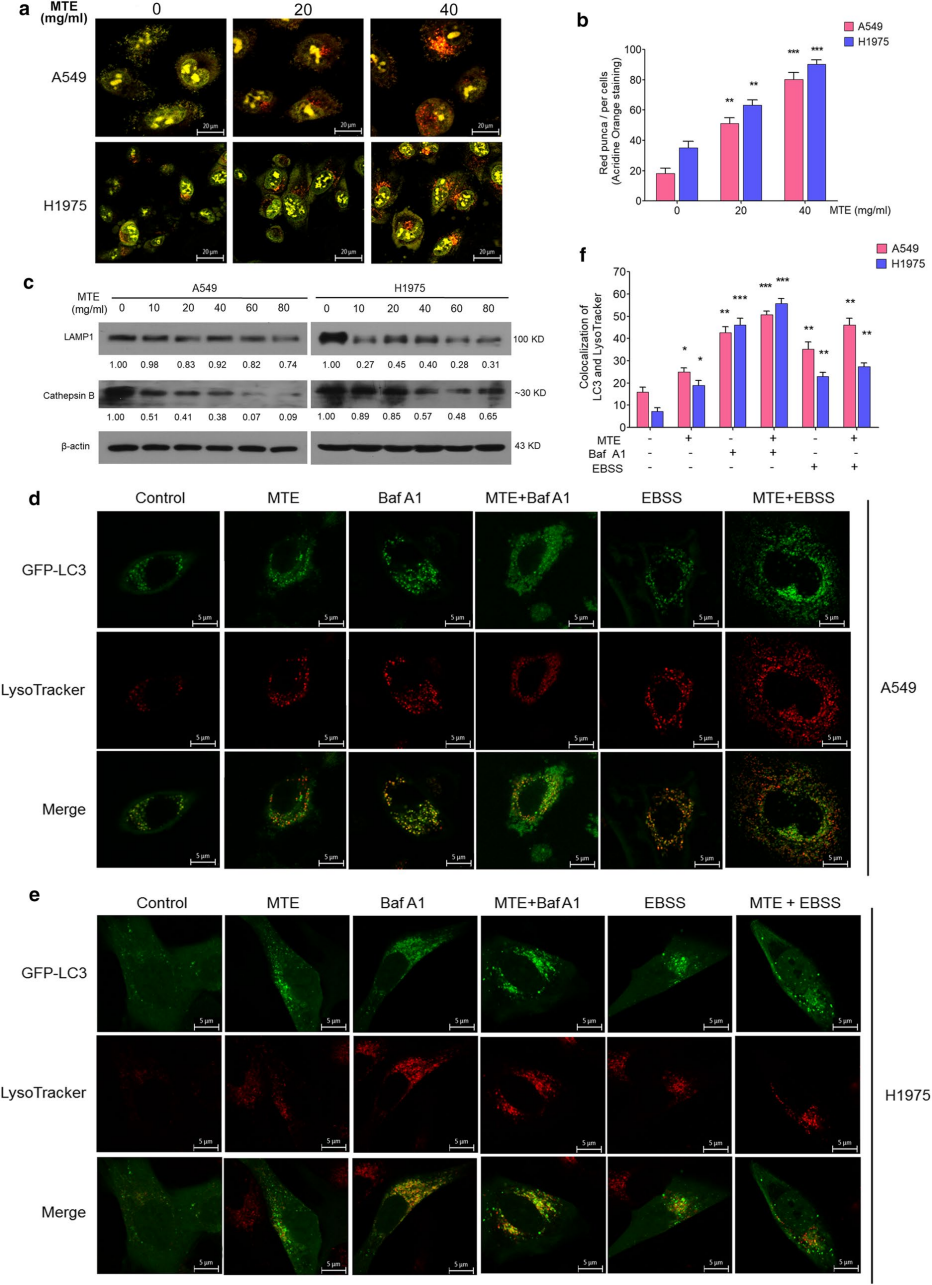
MTE suppressed autophagy by affecting lysosomal function. **a** Acidic vacuolar compartment in cells treated MTE with 0, 20 and 40 mg/ml (red puncta) were measured by acridine orange staining. **b** Numbers of red puncta in A549 and H1975 cells treated as in (**a**) were counted. Data represents mean ± SEM of at least 100 cells scored (*P < 0.05). **c** The protein level of LAMP1, Cathepsin B in treated cells were detected by Western blot, and protein expression levels were counted. Cells were treated with 0, 10, 20, 40, 60, 80 mg/ml MTE for 24 h. β-Actin was used as a loading control. **d**, **e** Colocalization of GFP-LC3 (green) and LysoTracker (red) were visualized on confocal microscope. GFP-LC3 stable A549 (**d**) and H1975 (**e**) cells were pretreated with 40 mg/ml MTE for 24 h following by Baf A1 (5 nM) or EBSS for another 4 h, and then incubated with LysoTracker for 90 min to be observed by confocal microscope. **f** Numbers of yellow (merge of green and red) puncta in cells treated as in (**d**, **e**) were counted. Data represents mean ± SEM of at least 100 cells scored. *P < 0.05, **P < 0.01, ***P < 0.005 vs control

In order to further investigate the influence of MTE on the late stage of autophagy, we focused on the lysosomal function, which is critical for the maturation of autophagosomes and the degradation of their contents. The intralysosomal pH plays an important role in affecting the Cathepsin enzymatic activity and lysosomal functions. So, firstly we performed AO staining to visualize acidic vesicles after MTE treatment. AO is a fluorescent weak base that fluoresced bright red when accumulating in acidic compartments such as autolysosome and lysosome, whereas fluoresced bright green in cytoplasm and nucleolus [23]. As shown in Fig. 4a, b, MTE treatment for 6 h resulted in visible and increased bright red vacuoles compared with control groups both in A549 and H1975 cells, suggesting MTE treatment significantly decreased intralysosomal pH. Next, we tested whether MTE can affect lysosomal function through detecting LAMP1 and activated Cathepsin B by Western Blot. LAMP1 is located in lysosomal membrane involving in lysosomal motility, and Cathepsin B is one of the most important proteases inside lysosome [24]. As shown in Fig. 4c, MTE treatment reduced LAMP1 and Cathepsin B protein expression with increased MTE concentration, confirming that MTE impaired lysosomal function.

Furthermore, we examined the fusion between autophagosome and lysosome by monitoring LysoTracker to detect its colocalization with the autophagosomal marker LC3 in GFP-LC3 stable cells. Presence of MTE in GFP-LC3 stable cells led to an increase of GFP-LC3/LysoTracker colocalization compared to control cells (Fig. 4d for A549 and e for H1975, group 2 vs. group 1 in d–f). Such changes were similar to that cells treated with Baf A1 (Fig. 4d–f, group 4 vs. group 3). In EBSS treated A549 cells, GFP-LC3 punctation in MTE group was a little more colocalized with LysoTracker compare to control group, while no obvious change in H1975 cells (Fig. 4d–f, group 6 vs. group 5) was observed. Taken together, these results indicated that MTE impaired the fusion of autophagosome and lysosome, further confirming that MTE inhibits autophagy at the late stage in A549 and H1975 cells.

### ERK activation is required for apoptosis and autophagy regulation by MTE treatment

**Fig. 5:**
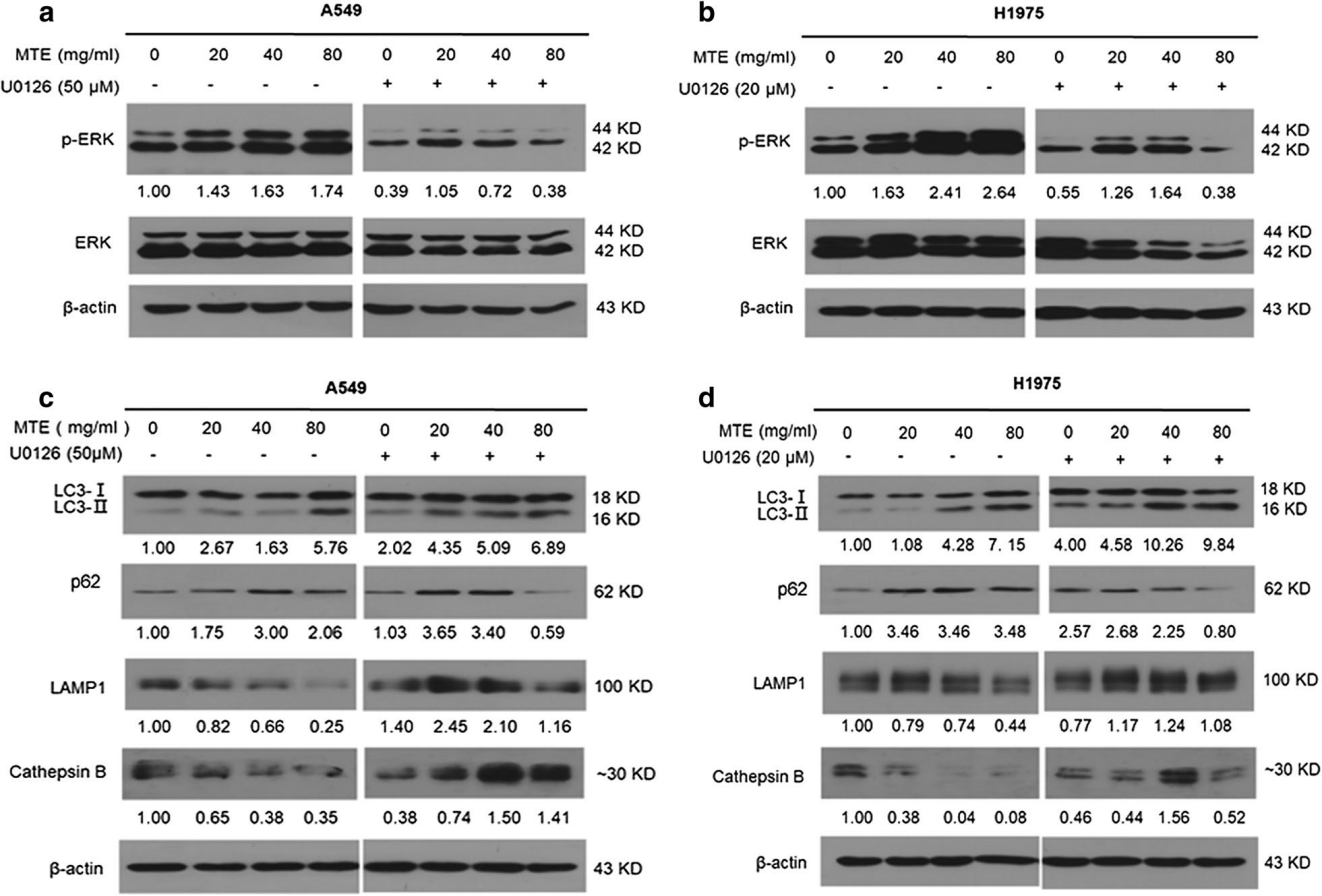
ERK activation is required for autophagy suppression by MTE treatment. A549 or H1975 cells were treated with MTE at concentration of 0, 20, 40, and 80 mg/ml for 24 h in the absence or presence of U0126. **a**, **b** The protein levels of ERK, p-ERK in treated cells determined by Western blot in A549 (**a**) and H1975 (**b**) cells. The ratio of p-ERK vs ERK protein levels in treated cells was counted. **c**, **d** The protein levels of LC3-II, p62, LAMP1 and Cathepsin B in treated cells were determined by Western blot in A549 (**c**) and H1975 (**d**) cells

It has been reported that both autophagy and apoptosis are regulated by the MEK/ERK pathway [25, 26]. Thus, we examined whether this pathway accounts for MTE caused cell apoptosis induction and autophagy inhibition. Western blot results in Fig. 5a, b (left panel) showed that MTE treatment upregulated phosphorylated ERK with a dose-dependent manner in both NSCLC cells. The role of ERK was further determined by using MEK/ERK inhibitor U0126, and we found that the activation of ERK by MTE was obviously attenuated via co-treatment with U0126 in A549 and H1975 cells (Fig. 5a, b, right panel). The above results showed that MTE activated MEK/ERK signaling in NSCLC.

Next, we examined the influence of ERK on autophagy associated molecules by MTE treatment. As shown in Fig. 5c, d, pre-treatment with U0126 deteriorated the autophagy inhibition caused by MTE, resulted in autophagy induction with increased LC3-II and decreased p62 in both cells. The function of lysosomes was damaged by MTE with down-regulated expression of LAMP1 and Cathepsin B. However, in presence of U0126, LAMP1 and Cathepsin B were upregulated, implying partly recovery of lysosomal function (Fig. 5c, d). These results demonstrated that MTE-caused autophagy inhibition was reversed by U0126, suggesting that MEK/ERK pathway contributed to the autophagy inhibition caused by MTE.

**Fig. 6:**
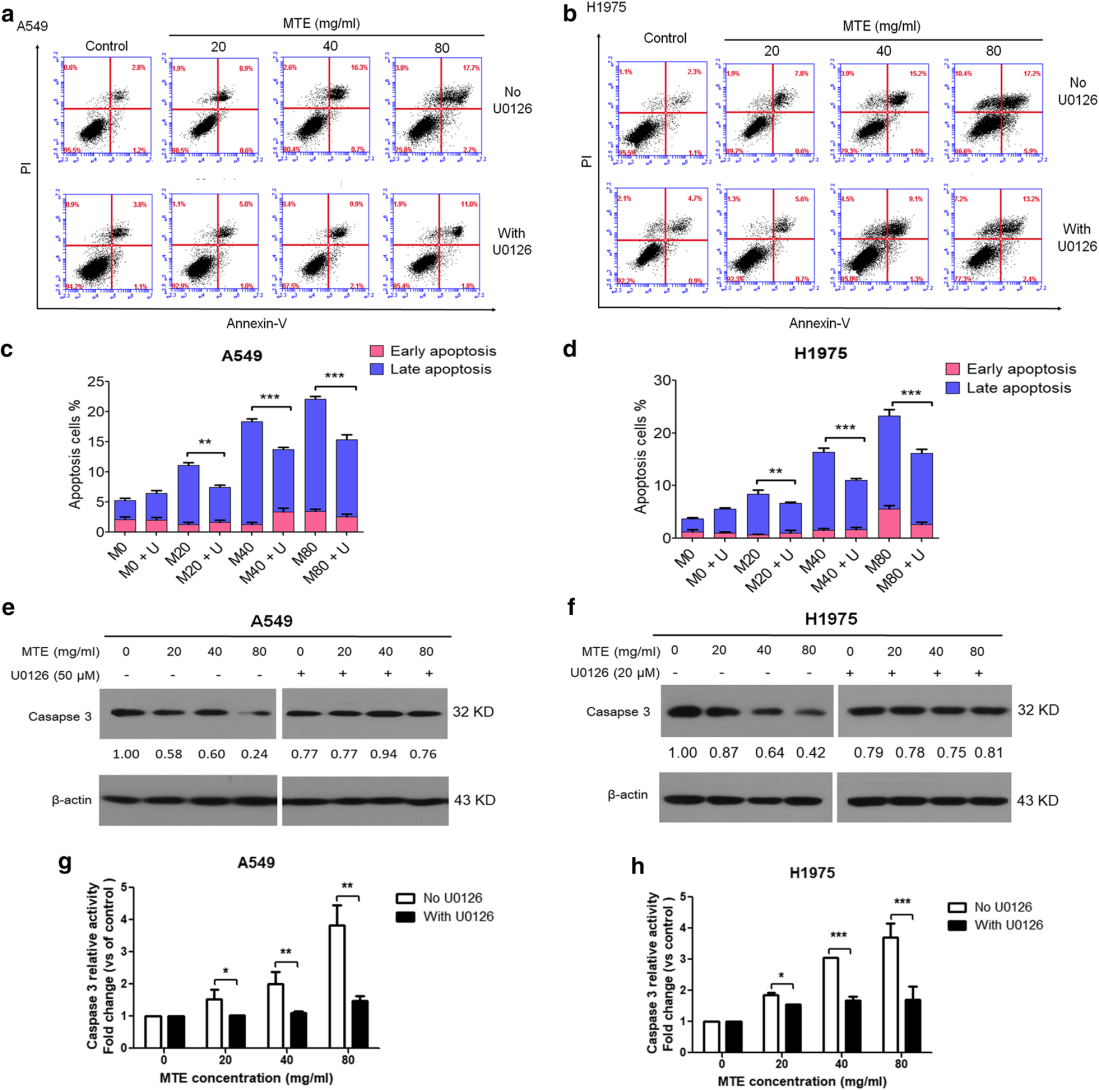
ERK activation is required for apoptosis induction by MTE treatment. **a**, **b** Apoptotic cells with indicated treatment were counted by Annexin V/PI assay. **c**, **d** The percentage of apoptotic cells with treatment was quantified. Early-stage apoptotic cells were shown in red, and late-stage apoptotic cells were shown in blue (M: MTE; U: U0126). **e**, **f** The protein level of Caspase 3 in treated cells was detected by Western blot, and the expression ratio was counted. Cells were treated with 20, 40, 80 mg/ml MTE for 24 h. **g**, **h** The caspase 3 activities in treated cells were detected. *P < 0.05, **P < 0.01, ***P < 0.005; Groups with U0126 vs groups without U0126

Finally, we evaluated the association between MTE-induced apoptosis and MEK/ERK pathway; and found cell apoptosis, especially late-stage apoptosis was significantly decreased in the presence of U0126. As shown in Fig. 6a–d, MTE-caused late apoptotic A549 cells ranged from 4.5 ± 0.7% to 12.7 ± 1.6% in U0126 pretreated group, while it was 3.3 ± 0.5% to 18.6 ± 0.8% in no U0126 group. The ratio of MTE-induced late apoptotic H1975 cells was from 4.7 ± 0.4% to 13.5 ± 1.3% in U0126 pretreated group, compared with 2.5 ± 0.5% to 17.7 ± 2.1% in no U0126 group. There is no obvious difference for the early apoptotic cells in each group. The results suggested that MTE-induced apoptosis was dramatically attenuated by U0126. As shown in Fig. 6e, f, MTE treatment relieved the protein levels of zymogens of Caspase 3 in U0126 group compared with no U0126 group in both cell lines. Caspase 3 activity induced by MTE was decreased significantly after pretreated with U0126 (Fig. 6g, h). These data indicated that ERK activation is required for MTE-induced apoptosis.

## Discussion

Studies showed C21 steroidal glycosides are major components in *M. tenacissima*. Compounds such as Tenacigenoside A [13], 11α-*O*-benzoyl-12β-*O*-acetyl tenacigenin B [27], tenacissoside C, tenacissoside B, tenacissoside C, Tenacissoside I and marsdenoside K [28] etc. are demonstrated to possess anti-cancer activity. According to our previous HPLC–MS analysis, 13 compounds including most of compounds mentioned above were identified from MTE by HPLC–MS analysis [12]. In recent years, *M. tenacissima* has attracted extensive interest in cancer research area with multiple effects, such as inhibiting tumor growth and angiogenesis, reversing anti-tumor drug resistance [12, 14, 17]. However, the reasons why MTE treatment resulted in the inhibition on cancer cell growth still remain largely unknown. In the present study, we found MTE significantly induced cell apoptosis, suppressed cell autophagy through impairing lysosomes function in A549 and H1975 NSCLC cells. Our results also indicated that ERK may mediate autophagy inhibition and apoptosis induction effect of MTE in NSCLC cells.

Programmed forms of cell death pathway at least include apoptosis and autophagy. Apoptosis is a physiological process to eliminate damaged, mutant or aged cells to maintain cellular homeostasis in normal tissue [29]. The inhibition of apoptosis is regarded as one of the hallmarks of cancer [3], and apoptosis-inducing has been exploited as an indispensable anticancer therapeutic strategy. Approaches targeting apoptotic pathway can result in cancer cell death, increasing sensitivity to current treatments or reversing drug resistance, thus may bring promising clinical benefits. Till now, different apoptosis targeted therapies have entered clinical trials for efficacy evaluation in various tumor types including lung cancer [30]. In the present study, MTE induced significant cell apoptosis in both A549 and H1975 cells along with Caspase 3 activation. Although cleaved PARP was not observed in A549 cells with MTE treatment, remarkable apoptotic cells presented after stained with Annexin V-FITC for flow cytometry analysis. The above results indicated that apoptosis-inducing may contribute to the cell death caused by MTE treatment.

Autophagy has complicated functions on cell death, as it may promote or inhibit cell death under certain circumstances. Although autophagy may offer tumor suppressive function in some conditions [31], it is mainly a cytoprotective process to facilitate cancer cells survive under stressful environments [32]. Studies showed that autophagy suppression in NSCLC cells resulted in cell proliferation suppression [33] and cell apoptosis increase [34]. In addition, constitutive activation of autophagy is also associated with anti-cancer therapeutic resistance [35], and inhibiting autophagy may overcome drug resistance in tumors [36]. Therefore, targeting autophagy is considered as a potential therapeutic strategy for cancer treatment.

LC3-II and p62 serve as marker of autophagic flux. The level of p62 increased when autophagy inhibition occurred; and decreased when autophagy is induced. In our study, MTE treatment caused accumulation of both LC3-II and p62, which means the substrate degradation was blocked and autophagic flux was impaired. The autophagy inhibitory effect of MTE was further confirmed by adding autophagy inhibitor Baf A1 and autophagy inducer EBSS. Next, GFP-LC3 stable NSCLC cells labeled with LysoTracker showed MTE suppressed the fusion of lysosomes with autophagosome at the late stage. This effect was further confirmed by detecting lysosomal marker LAMP1 and lysosomal protease Cathepsin B, indicating MTE impaired lysosomal function. Consistent with our results, other study demonstrated that inhibition of the fusion between lysosomes and autophagosomes leading to accumulated LC3-II and increased acidic vacuolar compartment [37]. Our results demonstrated that MTE can target both apoptosis and autophagy leading to NSCLC cells death. In fact, the molecular connections exist between apoptosis and autophagy, and some regulators are shared to maintain a subtle and complicated balance with each other [38–40]. ERK is a crucial molecule to control diverse cell responses including proliferation, migration, and differentiation [25]. High levels of ERK has been found in many malignant tumors, but ERK activation is not always correlated with cell survival protection, it can also interplay with cell death including apoptosis, autophagy, and senescence [25, 26]. Growing evidence demonstrated that activated ERK has positive contribution to cancer treatment, such as induced cell apoptosis and cell death [41, 42]. BPIQ, a synthetic quinoline analog, upregulated ERK phosphorylation leading to H1299 cell death, and this can be abrogated by ERK inhibitor [43]. In consistent with other studies, our results demonstrated that ERK activation plays important roles in drug-induced cancer cells apoptotic death.

Accumulated evidence demonstrated activated ERK is also involved in autophagic cell death [26]. 8-CEPQ, a novel quercetin derivative, inhibited colon cancer cell growth by inducing autophagic cell death through ERK activation [44]. Tan IIA induces autophagic cell death via activation of AMPK and ERK in KBM-5 cells, and ERK inhibitor PD184352 suppressed LC3-II expression induced by Tan IIA [45]. Rhuscoriaria induced autophagic cell death through p38 and ERK1/2 activation in breast cancer cells [46]. All the above studies link ERK activation with autophagic cell death. In our study, MEK/ERK inhibitor U0126 effectively abrogated the impaired autophagy flux caused by MTE. Taken together, our results revealed the effect of MTE on cell apoptosis-induction and autophagy-inhibition can partly ascribe to ERK activation.

## Conclusion

A Chinese herb preparation, MTE, induced significant cell apoptosis and impaired the fusion of lysosomes with autophagosome in NSCLC cells A549 and H1975. The molecule ERK, which links the crosstalk between apoptosis and autophagy, partly accounts for the underlying mechanisms of cell death caused by MTE. As cancer has complex networks of signaling pathways, thus multiple targeting autophagy and apoptosis by Chinese medicine may shed some light on the way for NSCLC cancer treatment.

## Additional file

**Additional file 1: Fig. S1.** Mitochondrial associated proteins were involved in MTE-induced apoptosis. (**A**, **B**) The protein level of Bcl-2 alpha and Bax in treated A549 cells (**A**) and H1975 cells (**B**) were detected by Western blot, and the ratio of protein levels treated was counted. Cells were treated with 0, 10, 20, 40, 60, 80 mg/ml MTE for 24 h.

## Abbreviations

SCLC: small-cell lung cancer
NSCLC: non-small cell lung cancer
MTE: water extract of *Marsdenia tenacissima*
EBSS: Earle’s balanced salt solution
MTT: 3-(4,5-dimethyl-2-thiazolyl)-2,5-diphenyl-2-*H*-tetrazolium bromide
PARP: poly (ADP-ribose) polymerase
OD: optical density
PAGE: polyacrylamide gel electrophoresis
BSA: bovine serum albumin
PBS: phosphate-buffered saline
AO: acridine orange
SEM: standard error of mean
IC50: half maximal inhibitory concentration
Baf A1: Bafilomycin A1
DAPI: 2-(4-amidinophenyl)-6-indolecarbamidine dihydrochloride
LAMP1: lysosome-associated membrane protein 1
