# Supplementary figures and images for: Marsdenia tenacissima extract induces apoptosis and suppresses autophagy through ERK activation in lung cancer cells
Source: Cancer Cell Int. 2018 Sep 27;18:149. doi: 10.1186/s12935-018-0646-4 (PMC6161462; doi:10.1186/s12935-018-0646-4)

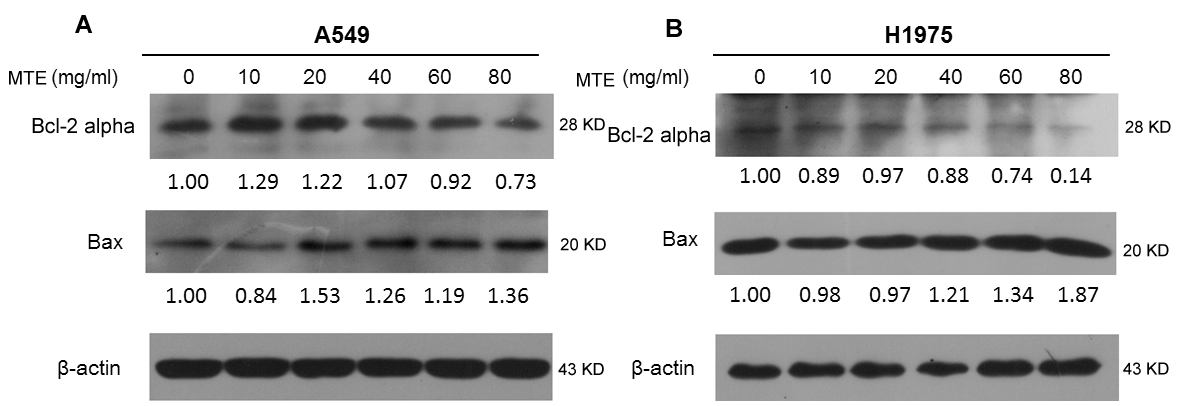

Supplement: Supplementary file 1 — Additional file 1: Fig. S1. Mitochondrial associated proteins were involved in MTE-induced apoptosis. (A, B) The protein level of Bcl-2 alpha and Bax in treated A549 cells (A) and H1975 cells (B) were detected by Western blot, and the ratio of protein levels treated was counted. Cells were treated with 0, 10, 20, 40, 60, 80 mg/ml MTE for 24 h. [file 12935_2018_646_MOESM1_ESM.tif]
